# State‐space modeling reveals habitat perception of a small terrestrial mammal in a fragmented landscape

**DOI:** 10.1002/ece3.5519

**Published:** 2019-08-16

**Authors:** Riana Gardiner, Rowena Hamer, Vianey Leos‐Barajas, Cesar Peñaherrera‐Palma, Menna E. Jones, Chris Johnson

**Affiliations:** ^1^ School of Natural Sciences University of Tasmania Hobart TAS Australia; ^2^ Department of Statistics Iowa State University Ames IA USA; ^3^ Department of Business Administration and Economics Bielefeld University Bielefeld Germany; ^4^ Pontifical Catholic University of Ecuador Manabí Ecuador

**Keywords:** conservation, fragmentation, Hidden Markov Models, management, movement ecology, restoration

## Abstract

1. Habitat loss is a major cause of species loss and is expected to increase. Loss of habitat is often associated with fragmentation of remaining habitat. Whether species can persist in fragmented landscapes may depend on their movement behavior, which determines their capability to respond flexibility to changes in habitat structure and spatial distribution of patches.
2. Movement is frequently generalized to describe a total area used, or segmented to highlight resource use, often overlooking finer‐scale individual behaviors. We applied hidden Markov models (HMM) to movement data from 26 eastern bettongs (*Bettongia gaimardi*) in fragmented landscapes. HMMs are able to identify distinct behavior states associated with different movement patterns and discover how these behaviors are associated with habitat features.
3. Three distinct behavior states were identified and interpreted as denning, foraging, and fast‐traveling. The probability of occurrence of each state, and of transitions between them, was predicted by variation in tree‐canopy cover and understorey vegetation density. Denning was associated with woodland with low canopy cover but high vegetation density, foraging with high canopy cover but low vegetation density, and fast‐traveling with low canopy cover and low vegetation density.
4. Bettongs did move outside woodland patches, often fast‐traveling through pasture and using smaller stands of trees as stepping stones between neighboring patches. Males were more likely to fast‐travel and venture outside woodlands patches, while females concentrated their movement within woodland patches.
5. *Synthesis and applications*: Our work demonstrates the value of using animal movement to understand how animals respond to variation in habitat structure, including fragmentation. Analysis using HMMs was able to characterize distinct habitat types needed for foraging and denning, and identify landscape features that facilitate movement between patches. Future work should extend the use of individual movement analyses to guide management of fragmented habitat in ways that support persistence of species potentially threatened by habitat loss.

## 1 INTRODUCTION

Human activities have caused loss and fragmentation of habitat in many parts of the world, restricting species to smaller and more degraded areas of their natural habitat and thereby contributing to global decline of biodiversity (Maxwell, Fuller, Brooks, & Watson, 2016). Management of animals threatened by habitat fragmentation often attempt to preserve or restore habitat by planting native vegetation, with the assumption that the resultant structure is suitable for the animals. These efforts can fail if habitat elements needed by the target species are not provided (Palmer, Ambrose, & Poff, 1997; Peipoch, Brauns, Hauer, Weitere, & Valett, 2015). Effective restoration therefore requires fine‐scaled understanding of how animals respond to details of habitat (Allen & Singh, 2016; Browning et al., 2018; McClintock, London, Cameron, & Boveng, 2017). Studies of animal movements are a powerful tool to provide this understanding. Movement patterns reflect short‐term behavioral decisions made by animals in response to their environment and can reveal which elements of the environment most affect habitat selection and should therefore be the focus of management (Jones & Davidson, 2016; Nathan et al., 2008).

In modified landscapes, the abundance, structure, and quality of resources are not equally distributed across patches. Separation of patches by distances greater than animals can normally cross hinders dispersal ability, isolating populations, decreasing gene flow, and ultimately increasing the risk of local extinctions (Crooks et al., 2017; Dixo, Metzger, Morgante, & Zamudio, 2009; Franzén & Nilsson, 2010). Moreover, fragmentation is often accompanied by habitat degradation. In lower quality patches, animals may be less likely to find the habitat resources they need. In both instances, increased fragmentation and loss will reduce the fitness and persistence of species dependent on the habitat type that has been reduced (Niebuhr et al., 2015; Roques & Stoica, 2007). Identifying characteristics of movement patterns of individual animals can be useful in revealing crucial attributes of habitat and quantifying the effects of distance between patches on isolation of local populations.

Animal movement is often quantified by describing the total area occupied and describing habitat features encompassed by a set of locations or segments of movement to determine area requirements and broad habitat preference. Technological and modeling advances have made it possible to collect more finely resolved data on the movement paths of individuals and to use the density and distribution of speeds and turning angles along those movement paths to infer behavioral states (Phillips, Patterson, Leroy, Pilling, & Nicol, 2015). For example, more tortuous angles and smaller intervals between successive locations (i.e., “steps”) may indicate foraging, or occupation of preferred habitat. Longer steps and smaller angles can indicate transit through less favorable habitat (Maciel & Lutscher, 2013; Osbourn, Connette, & Semlitsch, 2014), as seen in elephants (Duffy, Dai, Shannon, Slotow, & Page, 2011) and caribou (Avgar, Mosser, Brown, & Fryxell, 2013). Transitions between behavior states are also important in identifying the external factors that govern movement. Improvements in technology have made it possible to acquire large and finely resolved datasets on individual movements (Tucker et al., 2018) that are otherwise difficult to observe.

The eastern bettong (*Bettongia gaimardi*) is a member of the marsupial Family Potoroidae and weighs approximately 1.5 kg. Despite being polygynous the species is not territorial and does not display any sexual dimorphism. It is both a keystone species and ecosystem engineer because, like other potoroid marsupials, it disperses the spores of hypogeal fungi and modifies soil conditions as a result of digging for fungi, providing benefits for woodland health (Claridge, 2002; Fleming et al., 2014; Johnson, 1996; Vernes & Pope, 2001). The species was formerly distributed across the eastern half of Australia, but invasive predators caused extinction on the mainland early in the twentieth century (Johnson, 2006).

The remaining wild population of the eastern bettongs occurs in the eastern half of Tasmania. Much of the woodland and forest habitat of the eastern bettong in this region has been converted for agriculture, and woodland remnants are highly fragmented, especially in the intensively farmed bioregion of the Midlands which forms the core of the bettong's distribution. While the eastern bettong is a woodland specialist with large individual area requirements, it is able to persist in fragmented landscapes (at low population density) provided that a sufficient total area of habitat is available in the local landscape (Gardiner, Bain, Hamer, Jones, & Johnson, 2018). Persistence under these circumstances is likely to be strongly affected by the movement behavior of individuals, which allow them to gain access to the habitat area that they require.

In this study we apply HMMs, a form of state‐space modeling, to GPS tracking data on eastern bettongs to determine how individuals move in a landscape where their woodland habitat is fragmented by land clearance for agriculture. We use HMMs to categorize behavioral states from movement data and identify habitat attributes that influence transitions between those states. The eastern bettong is a nocturnal species, building nests in concealing vegetation in which to den during the day. Therefore, we expected them to concentrate their denning in woodland with denser vegetation. Previous studies have shown that higher stem density of canopy and midstorey trees (or saplings of canopy trees) is an attribute of preferred habitat, probably because it is associated with higher production of the ectomycorrhizal fungi on which the bettong feeds (Gardiner et al., 2018; Johnson, 1994b). Therefore, we suggest that the species is likely to concentrate its foraging in woodland areas with higher stem density and therefore higher canopy cover. Lastly, we predict that when vegetation cover is low or absent, such as in open pasture, bettongs are likely to travel faster as they are more likely to be exposed to predators, and they are likely to use such habitat only for transit between habitat patches. Thus, we tested whether the percent of canopy cover, vegetation density and distance to woodland edge influenced behavioral transitions.

## 2 METHODS

Animal ethics approval was obtained from the University of Tasmania (permit A14879) and the Department of Primary Industries, Parks, Water and Environment (permit: FA15118).

### 2.1 Study area

The Midlands covers 7,760 km^2^ of the eastern central area of Tasmania, Australia. The region is moderately dry (annual rainfall is typically 450–500 mm), with mean winter average temperature reaching 5°C and summer averages of 20°C. The region hosts a number of species of endemic fauna and flora, including a suite of marsupials that are threatened or extinct outside Tasmania. The natural vegetation of the region is grassland and open woodland, but over the last 200 years much of this has been converted to improved pasture or cropland, such that only 10% of the original woodland and 3% of the original grassland remains (Jones & Davidson, 2016). Most remnants of original habitat are on private or protected property, and are often fragmented by roads, grazing pasture or plantations.

**Figure 1:**
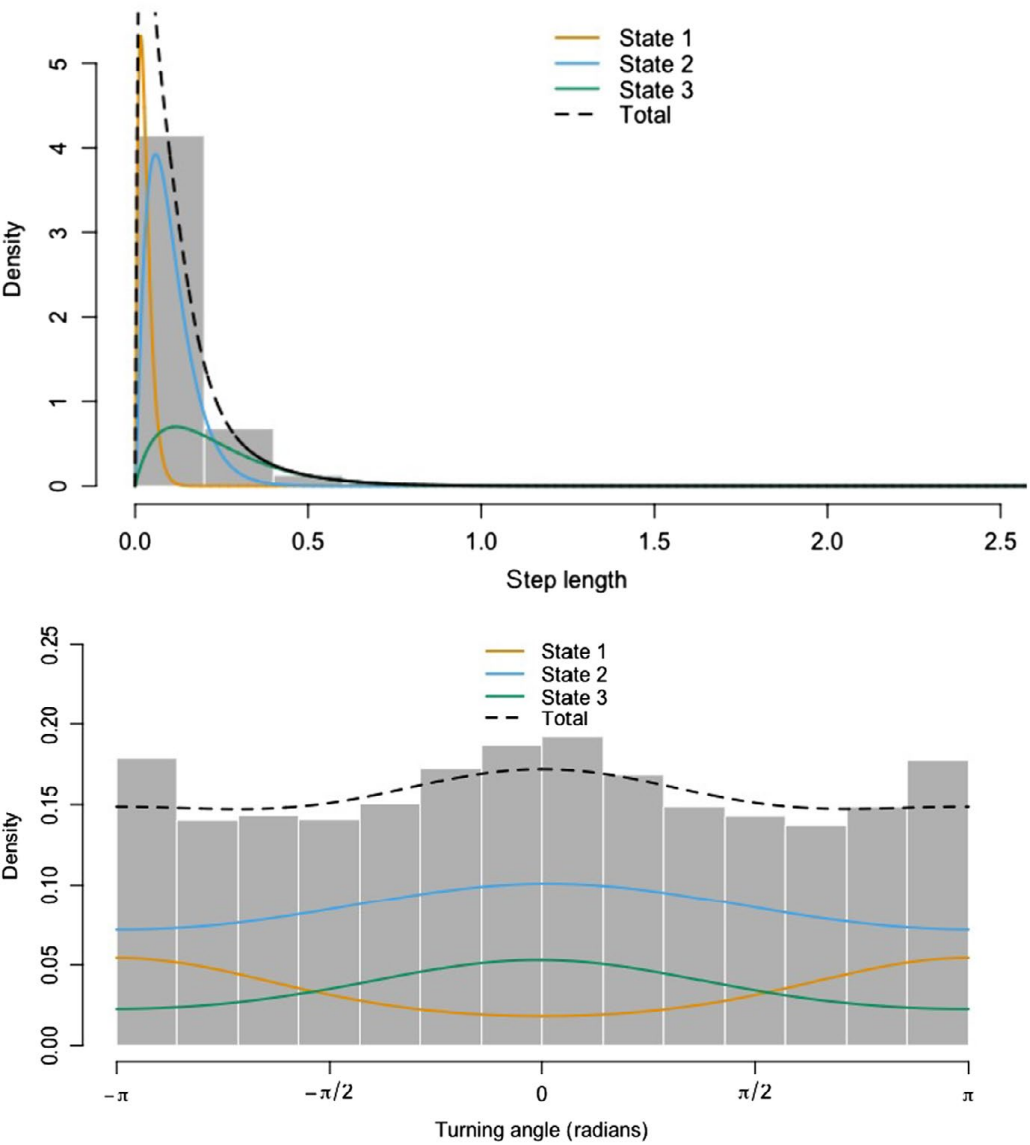
Histogram depicting the density of step lengths and turning angle distributions derived from a three‐state model for all tracked individuals

We studied movements of eastern bettongs at three sites that differed in amount of remnant canopy cover and degree of fragmentation. Woodlands at each site are broadly described as dry sclerophyll woodland, dominated by *Eucalyptus amygdalina* as the overstorey species, *Acacia dealbata* in the midstorey and with a patchy distribution of *Lomandra longifolia* (mat rushes) and *Pteridium esculentum* (bracken fern) in the ground level layer. Previous studies described habitat quality and the amount of fragmentation at each site (Gardiner et al., 2018). Site 1 is the least fragmented, site 2 is moderately fragmented, and site 3 is the most fragmented (Figure 1). Site quality has previously been measured as stem density of regenerating overstorey species (Gardiner et al., 2018). Of the three sites, site 1 is considered to be of lower habitat quality than the more fragmented site 2 and 3 (Appendix S1).

### 2.2 Trapping and tracking

We trapped Eastern bettongs between March 2016 and May 2017 (Table 1). Trapping was carried out for 3–5 days a week for 3 weeks at each site. Traps were wire cage traps (Mascot Wire Works), baited with balls of peanut butter and rolled oats, set along transects running through the middle of woodland patches at 150 m intervals. Upon capture, each individual bettong was PIT‐tagged for identification, sexed, and weighed. Animals were collared whether they weighed more than 1.5 kg, to ensure that only mature adults received collars. Each collar included a dual G10 UltraLITE GPS logger and VHF transmitter (Advanced Telemetry Solutions) with an average accuracy of ±20 m. Collars were deployed for approximately one month on each individual, and the GPS logger was set to record fixes every 15 min between 1600 and 0600 hr. VHF tracking was carried out regularly to ensure collars were still functioning and still on the animal. All fixes recorded the night an individual was fitted with a collar and the collar retrieved were removed from the analysis. We also mapped tracks by day using ArcGIS tool “Tracking Analyst” and removed fixes that were beyond an animal's usual range such as in water bodies or were clearly beyond the distribution of points.

**Table 1:**
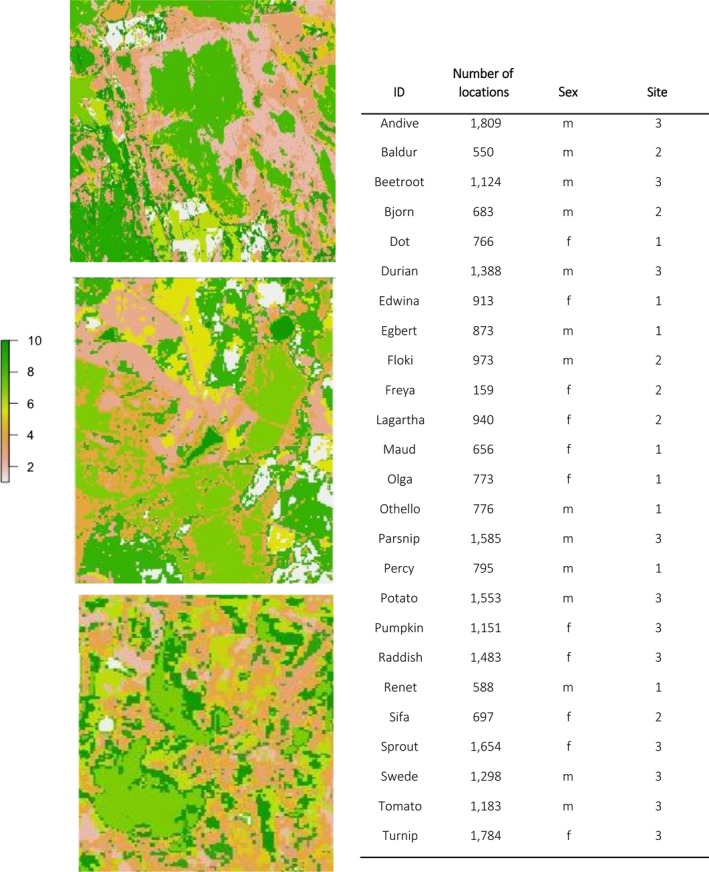
Tracking of eastern bettongs at the three different sites in the Midlands bioregion of Tasmania, Australia. Each site differed in the size, configuration, and quality of habitat, site 1 being low in fragmentation, site 2 medium, and site 3 high fragmentation. The sites are represented using unsupervised *K*‐mean values of Landsat imagery to classify vegetation density between 0 and 10 mean clusters

### 2.3 Site attributes

We identified habitat types associated with each relocation point by overlaying tracking data onto the state‐wide vegetation mapping dataset TASVEG 3.0 (Department of Primary Industries, 2013) using ArcGIS 10.5. We then calculated the Euclidean distance of points to the edge of the closest area of woodland. We also extracted values of percent canopy cover from TERN Auscover forest layers (http://data.auscover.org.au/xwiki/bin/view/Product+pages/Persistent+Green-Vegetation+Fraction).

We wanted to highlight differences in vegetation density (structure) particularly within woodland sites. Using package raster (Hijmans et al., 2017) in R, we extracted infrared bands from Landsat 5 raster layers (https://landsat.usgs.gov/landsat-in-action). We highlighted differences in density of vegetation, by first calculating values of the Normalized Difference Vegetation Index (NDVI). We then used unsupervised *K* means classifications on NDVI layers to quantify vegetation densities. The *K*‐mean algorithm classifies pixels based on the distances from cluster means (Lu & Weng, 2007), higher values indicating denser vegetation, lower values indicating open habitat to bare pasture. We compared the *K*‐mean values to Google Earth imagery, as well as researchers' knowledge of the site, to verify that values were representative.

### 2.4 Hidden Markov Models

Hidden Markov models (HMMs) allow observed movements to serve as a proxy for the underlying behavioral states of interest, and further infer spatial and temporal effects of switching between behavioral states (Leos‐Barajas et al., 2017; Patterson, Thomas, Wilcox, Ovaskainen, & Matthiopoulos, 2008). They assume a set of behaviors represented by movement are dependent on an unobserved state and can capture patterns found in movement data, which are translated as a proxy behavioral state. Further, their ability to manage autocorrelated and missing data and to utilize large datasets make them attractive to ecologists. To date, they have mostly been applied to wide‐ranging marine species (Franke, Caelli, Kuzyk, & Hudson, 2006; Hart, Mann, Coulson, Pettorelli, & Trathan, 2010; Towner et al., 2016).

Analysis of movement was carried out using the moveHMM package (Michelot, Langrock, & Patterson, 2016) in R 3.2.1 on all bettong tracks. Since HMMs are time‐series models, our data were formatted to represent each night of an animal as a single track which matched the normal activity times of bettongs (between 4 p.m. and 6 a.m.). Fixes which were not recorded due were coded as NAs. An HMM is defined by three components: the state‐dependent distributions (representing the values allocated to each behavioral state), the transition matrix (describing the evolution of the states over time), and the initial state distribution (probability of observing the states at the first time point; Zucchini, MacDonald, & Langrock, 2016).

We used gamma distributions for step lengths and von Mises distributions for turning angles. We modeled state transition probabilities as a function of site attributes. Vegetation density and sex were transformed into categorical variables, in which dummy variables (*K*−1) are added to the data as the probability of being observed at that time, as described by Michelot et al. (2016). Percent cover and distance to edge were treated as numerical variables. To examine how covariates affect state switching we computed stationary distributions as described by Patterson, Basson, Bravington, and Gunn (2009), to provide the marginal probability of a state at a given covariate value.

We assume independence between individuals' tracks and fit the HMM via maximum likelihood using direct maximization of the likelihood (Patterson et al., 2009). Models were run with single variables and additive combinations of covariates and ranked using the AIC criterion. For each of the models, we considered a variety of initial starting values to ensure we found the global maximum. Finally, model goodness of fit was assessed by examining pseudo‐residuals.

## 3 RESULTS

We collected 26,156 locations from 26 individuals, including 14 males and 12 females at three sites (Table 1) with a mean number of 1,084 observations for males and 998 for females. First, we examined how many states could explain the movement displayed by testing 2 and 3‐state models. Choosing the appropriate number of states can be challenging (Pohle, Langrock, Beest, & Schmidt, 2017), as traditional use of AIC ranking will favor the model with more states, which was the case in this analysis. Following the suggestion of Pohle et al. (2017) for choosing the number of states, we inspected pseudo‐residuals for models fit for 2 and 3 states and found a better fit for 3 states. Moreover, the fitted gamma state‐dependent distributions showed three unique movement types, and an additional examination of the temporal structure of the data, it was visible there were three structures present (Table 2 and Figure 1).

**Table 2:** Likelihood and AIC values obtained from the Hidden Markov Models testing (a) feasibility of a three‐state model versus a two‐state model, (b) Habitat attributes tested to determine what drives transitions between states using a three‐state model

Model	Delta AIC	Log likelihood
3‐state	0	−10,300.02
2‐state	2,442.44	−11,531.24
VegIndex + cover+sex	0	−9,842.499
VegIndex + sex	8.24	−9,852.619
VegIndex + edge+sex	108.54	−9,896.768
VegIndex + cover	266.89	−9,981.944
VegIndex	267.88	−9,988.439
Cover + edge+sex	367.59	−10,068.3
VegIndex + edge	367.78	−10,032.39
Sex + edge	469.28	−10,125.14
Sex + cover	481.15	−10,131.07
Sex	591.45	−10,192.22
Cover	683.89	−10,238.44
Edge + cover	776.13	−10,278.57
Null	795.05	−10,300.02
Edge	991.22	−10,392.11

State 1 was characterized by concentrated space use, with very short step lengths and more tortuous movement indicated by larger turning angles. State 2 was characterized by short step lengths and turning angles smaller than state 1 and state 3 by long steps with straighter paths and strong directionality (Figure 1). We interpreted state 1 as denning given the similarity to distribution of locations provided by stationary test collars. State 2 was interpreted as foraging and state 3 as fast‐traveling. The average step length in each state was 28 m ± 0.17, 103 m ± 0.6, and 268 m ± 1.7 for denning (which likely comprised 20 m of mean location error when the collar is stationary), foraging and fast‐traveling, respectively.

**Figure 2:**
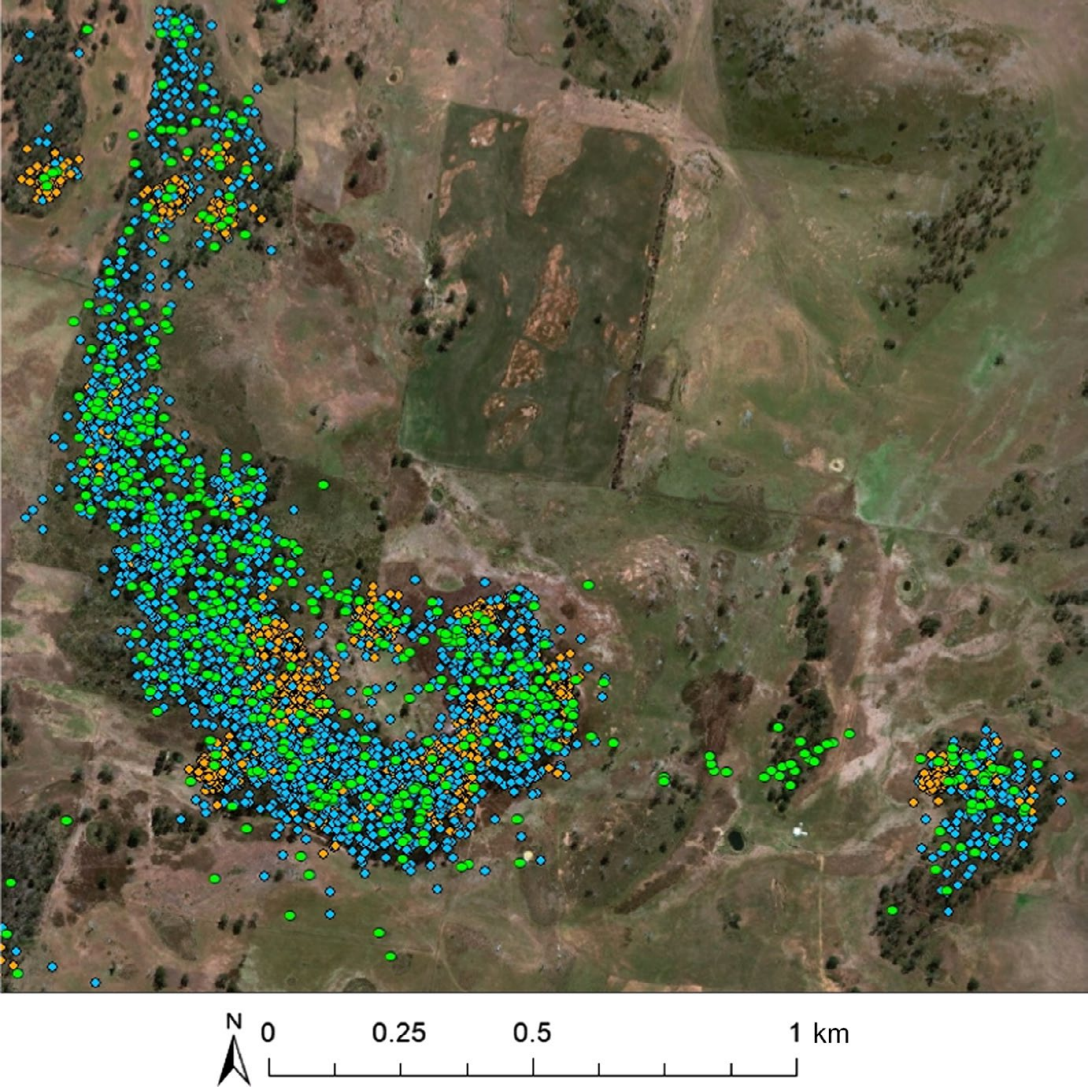
Example of bettong's locations, color coded by their corresponding state. Yellow is denning (state 1), blue is foraging (state 2), and green is fast‐traveling (state 3). This individual fast travelled across the matrix and used smaller stand of trees as stepping stones

Overlaying tracks on site maps showed that individuals used woodland more than any other vegetation type. Denning was displayed as a clumped pattern occurring in areas of dense vegetation, foraging extended throughout woodland patches within the individual's range and fast‐traveling included fast‐paced movement between patches or more open areas (example Figure 2). Across sites there was a difference in the proportion of time spent in different states. All animals spent a higher proportion of their nightly tracking period foraging than in any other state: 53% ,72%, and 66% of locations were represented foraging at sites 1 (low fragmentation), 2 (intermediate fragmentation), and 3 (high fragmentation) respectively. Fast‐traveling made up 40%, 22%, and 15% of time at the three sites and was most observed at the edge of the matrix or crossing to other patches, including small stands of trees (Figure 2). Denning made up the smallest proportion of locations, occurring only toward the end or very beginning of the nightly tracking periods.

Our models testing site attributes as predictors of transitions between states suggested that the model containing vegetation density and canopy cover, along with sex, were the best predictors (Table 2), out competing all other models by <8 delta AIC values and holding the lowest log‐likelihood of −9,842.499. The marginal probability of bettongs denning was highest in mid to dense vegetation but lower canopy cover. Probability of foraging was highest in mid to high canopy cover but lower understorey vegetation density. Fast‐traveling was most likely to occur in open to low understorey vegetation density and low canopy cover. The sex differences highlighted that males had a higher probability of transitioning to and from fast‐traveling than females, while females were more likely to remain foraging.

**Figure 3:**
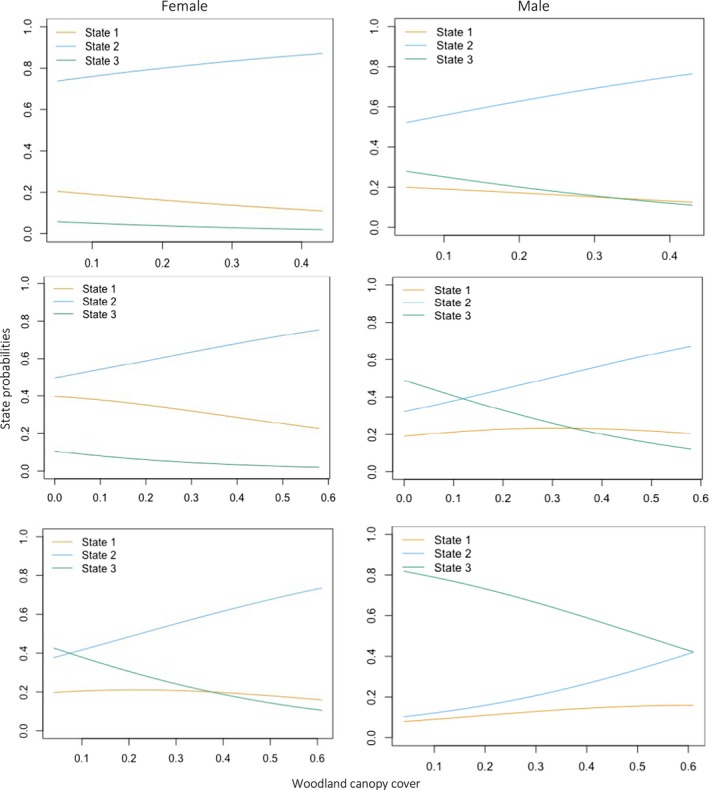
Example of stationary state probabilities of amount of cover for each sex. I show stationary probabilities for low (top), medium (middle), and high (bottom) woodland canopy cover

The transition probability matrix and stationary probabilities (Appendix S1) revealed there was a decreasing probability of transitioning between denning to foraging and traveling to foraging when understorey vegetation density was high (Figure 3). This suggests bettongs den in more dense vegetation and forage or fast‐travel through less dense vegetation. If canopy cover was high, there was a decreasing probability that bettongs would remain denning and/or transition from foraging to denning. Moreover, bettongs had higher likelihood of transitioning from denning to foraging and remain foraging or fast‐traveling with higher amount of canopy cover. This suggests bettongs will forage in higher canopy cover but move to lower canopy cover to den.

## 4 DISCUSSION

Our study applies state‐space modeling (specifically, hidden Markov models) to classify the behaviors of a small terrestrial vertebrate within a fragmented landscape. We were able to use data on movement pathways to identify three behavioral states and explain how sex and habitat features were associated with the occurrence of these behavior states. We interpreted behaviors as denning, foraging, and fast‐traveling and found that transitions between them were due to density of understorey vegetation and extent of canopy cover, subject to differences between the sexes. Our results provide insight into how eastern bettongs make decisions in relation to the characteristics of the landscape, in particular, their perception of the utility of the structure and configuration of vegetation. We discuss identified behavioral states and the knowledge gained for conservation purposes.

The eastern bettong is a woodland specialist with relatively large individual home ranges (90–200 ha). Previous studies exploring the responses of eastern bettongs to fragmentation and habitat characteristics highlighted the importance of habitat amount and quality in determining their occurrence (Gardiner et al., 2018) and the physical structure of home ranges (Gardiner, Proft, Comte, Jones, & Johnson in review). Our results from modeling movement pathways support these findings, but further identify the amount of cover and vegetation structure in the local environment as being important factors that explain variation in behavior of eastern bettongs. This is important information for management, highlighting which elements of habitat are being used and for what purpose.

Previous studies of habitat preferences in eastern bettongs suggested a lack of preference for particular floristics and vegetation structure (Johnson, 1994a); however, our study highlights behavioral responses to the understorey density of vegetation at ground level and to tree‐canopy cover. Denning was concentrated in areas with higher density of ground vegetation, provided by the presence of mat rushes *Lomandra longifolia* and bracken fern *Pteridium esculentum*, but with relatively low tree cover. Bettongs den in nests that they construct from material such as grass, fibrous bark, and bracken ferns. Their preferences for denning in dense vegetation may partly reflect availability of nesting material, but they are capable of transporting nesting material over quite long distances by carrying it in their prehensile tails, and they include material such as fibrous bark and fine tussock grasses that are not always available in the immediate vicinity of a nest. Bettongs may choose dense vegetation for nesting both to aid in concealment of the nest, particularly from aerial predators, and also to hide the animal's escape if it is disturbed while in the nest. Finding appropriate shelter is important for the survival of species, as this can include concealment from predators, rearing young and resting during inactive periods as seen across species such as hyenas (Singh, Gopalaswamy, & Karanth, 2010), lobsters (Heldt, 2013), and Pallas' cats (Ross, Kamnitzer, Munkhtsog, & Harris, 2010). The time spent denning was highest in site 3 than any other site, this could be an anti‐predator response where staying hidden for longer is more beneficial; however, this was not explicitly tested within this study. Our results suggest maintaining denser ground vegetation in woodland patches is important to provide denning resources for species fitness and survival.

Eastern bettongs foraged only in areas with canopy cover, which was expected for a woodland specialist. This can be explained by the fact that the species feeds mainly on the sporocarps of ectomycorrhizal fungi (Johnson, 1994b), which associate with the fine roots of woodland trees and shrubs. More open woodland are also associated with lower fertility soils, which are suitable conditions for ectomycorrhizal fungi networks, and can explain why foraging tends to occur further away from denser vegetation. Movement through denser vegetation is likely to be difficult where vegetation is often taller than the animal. On the other hand, pasture soils are frequently fertilized and nutrient‐rich, unsuitable for ectomycorrhizal growth (Wardle et al., 2004) and therefore not useful as a resource for bettongs (Taylor, 1992). The expansion of agriculture or encroachment of nutrients can ultimately affect soil conditions in woodland and directly affect foraging opportunities and can lead to large population losses (Runge, Martin, Possingham, Willis, & Fuller, 2014). Individuals from site 2—the smallest area of woodland—spent more time foraging than any other site, possibly because individuals may experience higher competition of resources and have to compensate for depleted resources. The retention of high‐quality soils and habitat is recurringly acknowledged as an important aspect for habitat use and persistence of species in fragmented landscapes (Fahrig, 2007a).

Lastly, bettongs were likely to travel fast in more open areas with less cover. Similar movement patterns are expected to be observed when animals are moving through lower quality or less preferred habitat, particularly open pasture. Overall, our results show that the eastern bettong has a strong dependency on woodland vegetation communities, as they use all the woodland patches within their range, further suggesting that the total amount of habitat within their range is important. Preserving the total amount of woodland habitat can therefore be an essential management method for species with higher mobility.

Within fragmented landscapes, the configuration of woodland patches varies, differing in sizes, shapes, and distances from core habitat and state of degradation. Our study depicts how behavioral states change in fragmented landscapes, as a result of the attributes of the landscape. In less favorable environments, species tend to spend more time and energy searching for resources (Fahrig, 2007a; Osbourn et al., 2014) and move at higher speeds (Braaker et al., 2014; Graves, Farley, Goldstein, & Servheen, 2007). Across our three sites, bettongs moved faster with longer step lengths when crossing lower quality (quantified as the density of regenerating stems, as an indication of regenerating and healthy woodland, Appendix S1) areas such as open spaces, roads, and gaps between woodland through pasture. Interestingly, bettongs from the least fragmented site (Site 1, Appendix S1) spent more time traveling fast than in the more fragmented sites. This could be a result of the woodland being of overall lower quality, combined with stressors such as the presence of grazing livestock, which are absent from the other two smaller and more fragmented sites, as these are strictly under covenant protection. Previous findings indicate that bettongs concentrate their home ranges in areas of higher quality (Gardiner et al., in review); this study further shows that concentrated movement—foraging and denning—occurs only in woodland, in areas of higher quality and usually within the larger remnant patches within a site. Similar findings have been reported in hares (Ullmann, Fischer, Pirhofer‐Walzl, Kramer‐Schadt, & Blaum, 2018) Increased time spent fast‐traveling in lower quality habitats can negatively influence the survival of species by increasing the cost of movement for little return. This highlights the importance of retaining native vegetation and quality to promote its use.

Interestingly, our tracking results showed eastern bettongs used isolated elements within the landscape, such as small patches and stands of trees as stepping stones when traveling fast between larger woodland patches (example, Figure 2). This suggests that these isolated elements, which may not constitute suitable woodland patches for foraging or denning, can be important for movement within fragmented landscapes. Moreover, this also suggests that smaller patches can contribute to the amount of habitat available within a bettong's range, which was similarly reported by Gardiner et al. (2018). Thus, movement for a mobile species is not hindered by fragmentation if there is enough total habitat and if the gaps between patches are not too large. If gaps are too large, species are likely to be restricted to smaller amounts of habitat and further influenced by edge effects. In this situation, therefore, restoring and retaining habitat, regardless of configuration, within a species range can be beneficial. Rather, habitat quality influences the frequency and type of movement. Low‐quality habitats are likely to incur greater fitness costs, possibly threatening the persistence of populations within remnants over time (Robertson & Hutto, 2006). Therefore, mitigating the impacts of degradation by including high‐quality resources, managing grazing pressures and retaining woodland becomes more important as the rate of fragmentation increases.

It is common in mammals for males and females to display different movement patterns, home ranges and area‐use characteristics according to the resources that are important to their reproductive success (Harestad & Bunnel, 1979). In mammals, males often have larger ranges to incorporate multiple females and avoid other males. Females are likely to concentrate their movement in areas of high food and shelter to meet their reproductive requirements (Lewis, O'Connell, Lewis, Campagna, & Hoelzel, 2006). In the eastern bettong, males were more likely to venture outside woodland in fragmented sites potentially to maintain access to multiple females, while females tended to concentrate their movement within woodland, spending most of their time foraging. Maintaining habitat connectivity, for example in the form of stepping stones, could therefore increase habitat links for movement and genetic connectivity at the population level, specifically to optimize male movement. These sex differences highlight how each sex perceives habitat and, as an extension, how they use it. Each sex tends to prioritize and use resources differently according to their resource requirements (Butler, Sawyer, & Losos, 2007; Niebuhr et al., 2015; Stokke & du Toit, 2000), therefore identifying and maintaining resources for successful reproduction and survival in both sexes is useful for management. We did not differentiate between females with different ages and stages of young, or dispersal life stages such as weaned juveniles; however, this could be useful information (Kokko & López‐Sepulcre, 2006) to manage essential habitat across all life stages.

In a rapidly changing world, it is fundamental to identify habitat resources and animal responses to modified landscapes. Movement patterns provide a finer‐scale understanding of how animals perceive their habitat (Browning et al., 2018). Using hidden Markov Models, we were able to achieve this for the eastern bettong in a fragmented landscape. Our analysis reflects similar findings in previous home‐range analyses (Gardiner et al., in review), but provide even finer‐scale information as to the attributes that drive movement patterns and where in the landscape different types of behaviors occur and therefore what is essential for managing habitat. Having the ability to map the distribution of behaviors opens avenues for mapping and quantifying specific areas and characteristics which can be used to guide conservation efforts. Our study further shows that movement models like HMMs could also be applied to species that are quite restricted in their habitat; however, whether these could work for even smaller species will rely on the resolution of tracking devices and complexity of habitat.

## CONFLICT OF INTEREST

None declared.

## AUTHOR CONTRIBUTION

RG, RH, VLB, CP, MJ, CJ, conceived the ideas and designed the methodology; RG collected the data; RG, VLB, and CP analyzed the data; RG, RH, VLB, MJ, and CJ led the writing of the manuscript. All authors contributed to the final manuscript for publication.

## Data Availability

Dryad: https://doi.org/10.5061/dryad.kk2sd41.
