# Supplementary material for: State‐space modeling reveals habitat perception of a small terrestrial mammal in a fragmented landscape
Source: Ecol Evol. 2019 Aug 16;9(17):9804–14. doi: 10.1002/ece3.5519 (PMC6745662; doi:10.1002/ece3.5519)
Supplement: Supplementary file 1 [file ECE3-9-9804-s001.docx]

**Supplementary**

| Site | Quality | Woodland | Nesting | Foraging | Travelling |
| --- | --- | --- | --- | --- | --- |
| 1 | 40 | 1290 | 0.07 | 0.53 | 0.4 |
| 2 | 106 | 85 | 0.06 | 0.72 | 0.22 |
| 3 | 117 | 157 | 0.19 | 0.66 | 0.15 |

S1: Characteristics of each site and the proportion of locations recorded at each site. Quality and Woodland are measurements in hectares.

| Density | 1 | 1 to 2 | 1 to 3 | 2 to 1 | 2 | 2 to 3 | 3 to 1 | 3 to 2 | 3 |
| --- | --- | --- | --- | --- | --- | --- | --- | --- | --- |
| 1 | 0.6948635 | 0.079374 | 0.225763 | 0.9623675 | 0.000010 | 0.037623 | 0.283802 | 0.000043 | 0.716155 |
| 2 | 0.6183489 | 0.2731791 | 0.108472 | 0.00014348 | 0.999808 | 4.83E-05 | 0.007609 | 0.9918672 | 0.000524 |
| 3 | 0.3176661 | 0.6201988 | 0.0621351 | 0.00036023 | 0.999312 | 0.000328 | 0.956746 | 0.0097162 | 0.033538 |
| 4 | 0.2935931 | 0.6766378 | 0.0297691 | 3.64E-05 | 0.999934 | 2.97E-05 | 0.001845 | 0.998038 | 0.000117 |
| 5 | 0.3665472 | 0.605207 | 0.0282458 | 0.00015641 | 0.999724 | 0.000119 | 0.004592 | 0.9950543 | 0.000353 |
| 6 | 0.6494062 | 0.2558017 | 0.0947922 | 3.53E-05 | 0.999856 | 0.000109 | 0.001773 | 0.9980011 | 0.000225 |
| 7 | 0.5889754 | 0.2804557 | 0.1305689 | 0.00016287 | 0.99967 | 0.000167 | 0.986234 | 0.0014334 | 0.012333 |
| 8 | 0.6666664 | 2.75E-08 | 0.3333335 | 0.00787591 | 0.989074 | 0.00305 | 0.004803 | 0.995062 | 0.000135 |
| 9 | 0.3752657 | 0.5079104 | 0.1168239 | 0.00023539 | 0.999374 | 0.000391 | 0.00312 | 0.9967986 | 8.17E-05 |
| female | 0.8420377 | 0.090853 | 0.067109 | 0.886034 | 0.06175 | 0.052216 | 0.8694474 | 0.0436639 | 0.0868887 |
| male | 0.4269653 | 0.505908 | 0.067127 | 0.44603 | 0.382043 | 0.171928 | 0.2411653 | 0.1567168 | 0.6021179 |
| cover | - | 0.951873 | -2.69535 | -1.34802 | - | -2.416253 | -1.451458 | 1.389433 | - |

Supplementary 2: Transition probability matrix given the density of vegetation (low to high: 1-10), sex and percent cover.


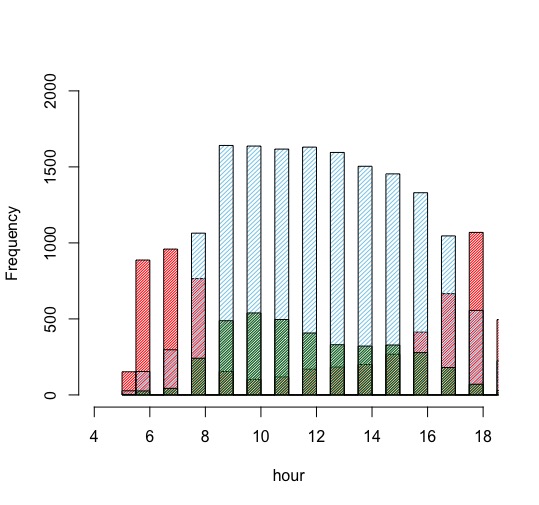


Supplementary 3: Frequency of states across time of active tracking between 4pm and 6am, each tick represent 2 hours. Red is State 1, Blue is state 2 and Green is state 3.
